# Current Status, Distribution, and Future Directions of Natural Products against Colorectal Cancer in Indonesia: A Systematic Review

**DOI:** 10.3390/molecules26164984

**Published:** 2021-08-17

**Authors:** Didi Nurhadi Illian, Ihsanul Hafiz, Okpri Meila, Ahmad Rusdan Handoyo Utomo, Arif Nuryawan, Gontar Alamsyah Siregar, Mohammad Basyuni

**Affiliations:** 1 Department of Pharmacy, Faculty of Mathematics and Natural Sciences, Universitas Syiah Kuala, Banda Aceh 23111, Indonesia; illian.didinurhadi@unsyiah.ac.id (D.N.I.); okprimeila@unsyiah.ac.id (O.M.); 2 Department of Pharmacology, Faculty of Pharmacy and Health, Institut Kesehatan Helvetia, Medan 20124, Indonesia; ihsanulhafiz@helvetia.ac.id; 3 Faculty of Medicine, Universitas YARSI, Jakarta 10510, Indonesia; ahmad.rusdan@yarsi.ac.id; 4 Department of Forestry, Faculty of Forestry, Universitas Sumatera Utara, Medan 20155, Indonesia; arif5@usu.ac.id; 5 Center of Excellence for Mangrove, Universitas Sumatera Utara, Medan 20155, Indonesia; gontar@usu.ac.id; 6 Department of Internal Medicine, Division of Gastroenterology-Hepatology, Faculty of Medicine, Universitas Sumatera Utara, Medan 20155, Indonesia

**Keywords:** colorectal cancer, WiDr, MTT, natural product, polyisoprenoids, terpenoids, Indonesia

## Abstract

In 2020, an estimated 19.3 million new cancer cases and nearly 10 million cancer deaths have occurred worldwide, with colorectal cancer ranking as the third most frequently diagnosed (10.0%). Several attempts have been conducted against cancer, including surgery, radiation, monoclonal antibodies, and chemotherapy. Many people choose natural products as alternatives against cancer. These products will not only help in human life preservation but also work as a source of up-to-date information, leading people away from incorrect information. We discuss the current status, distribution, and future implications of protecting populations with natural products as an alternative against colorectal cancer in Indonesia. Thirty-eight studies were included in this review for data extraction. The distribution of natural products in Indonesia that have potential activity against colorectal cancer cells was predominated by terpenoids, followed by phytosterols, phenolics, alkaloids, and polyisoprenoids. The type of cell line utilized in the cytotoxic activity analysis of natural products was the WiDr cell line, followed by HT-29 cells and HCT-116 cells. This review showed that MTT in vitro assay is a general method used to analyze the cytotoxic activity of a natural product against colorectal cancer cells, followed by other in vitro and in vivo methods. The systematic review provided predictions for several secondary metabolites to be utilized as an alternative treatment against colorectal cancer in Indonesia. It also might be a candidate for a future co-chemotherapy agent in safety, quality, and standardization. In addition, computational methods are being developed to predict the drug-likeness of compounds, thus, drug discovery is already on the road towards electronic research and development.

## 1. Introduction

Cancer is a deadly disease that has become a burden to everyone. There were 9.8 million cancer deaths reported in 2018. Colorectal cancer (CRC) is the third most common type of cancer globally, with 1.80 million cases, and it ranks second with the highest mortality in the world, i.e., 862,000 deaths [1]. The increase in CRC in developing countries is possibly due to an increase in the aging population, modern living habits, dietary habits, and an increase in risk factors for CRC, which include genetic diseases, smoking, alcohol, and lack of exercise. The percentage of CRC deaths in Indonesia in 2014 was 10% of the 103,000 CRC mortality rates in men and 8.5% of the 92,000 in women [2,3]. In Indonesia, CRC is an interesting case. For instance, the median age of colorectal cancer patients is younger than the western population. This means that productive young people are affected more, thus posing a heavy economic burden on their families [4,5,6].

Cancer treatment with chemotherapy agents is still an option, but the multi-drug resistance (MDR) mechanism has resulted in reduced efficacy of chemotherapy drugs [7]. The chemoprevention agents referred to here generally have the role of inhibiting tumor growth through cell cycle arrest mechanisms [8], stimulating apoptosis, or inhibiting the expression of proteins that play a role in MDR [9]. Various efforts are needed to develop new treatment methods for more effective therapy and prevention of degenerative diseases [10]. Alternative options such as the use of medicinal plants in the treatment of degenerative diseases can decrease any side effects [11]. Usually, lower effects may affect low efficacy, so there may be a trade-off. Therefore, there is a need to develop targeted therapy that uses super toxic plant-derived toxins as warheads that are to be conjugated to monoclonal antibodies targeting the CRC-specific antigens [12].

One of the strategies in the discovery and development of drugs for the prevention of degenerative diseases is by exploring natural products, especially plants, that have the potential to be sources of antioxidants. Plants are known to have an important role in drug discovery [13]. Natural products are secondary metabolites produced by plants, animals, and microorganisms in response to external stimulation such as changes in nutrition, infection, and competition [14].

## 2. Objective of the Review

The systematic review has the following primary review question:

- What evidence literature and data exist on the application of natural products from Indonesian plants in colorectal cancer?

The secondary review questions are:

- What are the available trends and study distribution of colorectal cancer in Indonesia?
- What are the used approaches and indicators for colorectal cancer derived from natural products in Indonesia?
- What are the challenges and gaps in the science and application of natural products in colorectal cancer in Indonesia?

## 3. The Scope of the Review

The systematic review is focused on studies that have reported the utilization of natural products in colorectal cancer in Indonesia. The data was gathered from published literature obtained through preselected bibliographic databases such as Scopus and PubMed, as well as search engines such as Google Scholar. To determine the scope of the review, this study used a globally applied standard approach for systematic review, by first defining the Population, Intervention, Comparator, and Outcome (PICO) of the review [15]. The detailed description of PICO used for this systematic review is as follows:

- *Population*: Colorectal cancer in Indonesia.
- *Intervention*: Any secondary metabolites and natural products used for colorectal cancer from Indonesian plants. These common approaches include in vitro and in vivo.
- *Comparator*: Any indicators used to monitor the success of anti-colorectal cancer activity by the use of natural products or secondary metabolites.
- *Outcome*: Inhibited colorectal cancer or no proliferation activity of colorectal cancer.

## 4. Methods

Most of the methodological steps in this systematic review follow previous systematic reviews on the health topic or pharmacy practice [16,17]. In addition, the reporting guidelines for systematic reviewers in the medical and health sciences are used in this systematic review report [18].

### 4.1. Literature Search

Before conducting a literature search in the bibliography database, we composed a search string by identifying the relevant keywords following the defined PICO. However, we only considered keywords from Population and Intervention categories to avoid the narrowed search string and reduce the number of papers. Literature searches in Scopus and PubMed were conducted by using the English search string, while we used both an English and Bahasa Indonesia search string for Google Scholar (see Table 1 and Table 2). To avoid the inclusion of further irrelevant studies in Google Scholar results, we only selected the first 50 publications, following the most relevant order.

### 4.2. Literature Screening

The study’s relevance was determined by using the inclusion criteria presented in Table 3. The studies must meet the following relevant criteria: population, intervention, comparator, and outcome of interest. After duplicates were removed from the search results, all studies went through a two-stage screening process at the combined title and abstract, and full-text levels three reviewers. All screening stages used predefined questions—formulated following PICO—to select which publications satisfy the scope of the review.

### 4.3. Reporting and Presentation

The reporting and presentation of the results of this systematic review followed a standardized reporting approach for the systematic review of health and pharmacy studies [16,17,18]. A database containing systematic review screening results is complementarily presented in addition to the report.

## 5. Results

**Figure 1:**
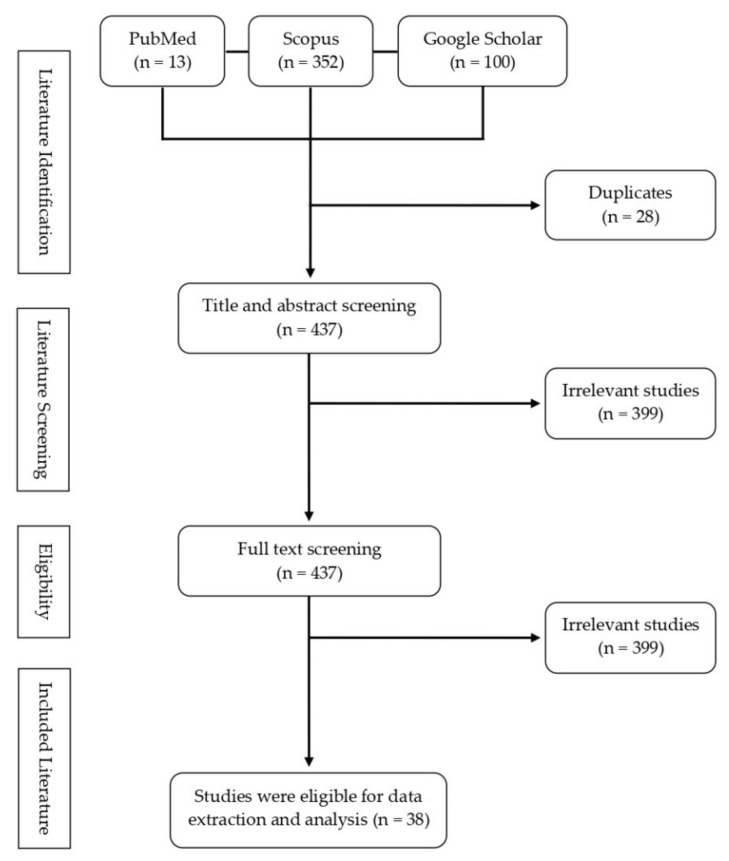
The PRISMA flow diagram showing the selection of eligible studies.

The PRISMA flow diagram of the publication screening is presented in Figure 1. Initially, 465 publications were identified through the systematic literature search string described in Table 1. Following the relevance of the initial papers to the defined research questions for this study, the literature screening, which consisted of title, abstract, and full-text screenings, resulted in 38 final papers for further data extraction. Overall, the screening processes only included 10% of the initially identified papers in Scopus, PubMed, and Google Scholar publication databases. The literature search results from major scholar databases such as Scopus as well as Google Scholar suggested the approximate publications that were recorded out of publications with duplicates (Figure 1). The cut-off date of the literature search was 13 February 2021.

As presented in Table 4, the research publication on colorectal cancer based on natural products derived from Indonesian plants has increased since 2008. The trend of publications seems to have increased significantly, starting in 2016, and it was the highest in 2018. Afterward, the positive trend continued until 2020, with the most publications in the form of journal articles.

Table 5 exhibits that the distribution of natural products having the potential activity against colorectal cancer cells in Indonesia is dominated by terpenoids, followed by phytosterols, phenolics, alkaloids, and polyisoprenoids. The type of cell line that is most often utilized in the cytotoxic activity analysis of natural products against colorectal cancer cells is the WiDr cell line (followed by HT-29 cells and HCT-116 cells). WiDr cells are human colorectal cancer cells isolated from the colon of a 78-year-old woman, a derivative of other colorectal cancer cells, namely HT-29 cells [19]. This data led to the MTT in vitro assay as a general method that was used to analyze the cytotoxic activity of a natural product against colorectal cancer cells, followed by other in vitro and in vivo methods. Commonly known anticancer methods include the MTT assay method, apoptosis, enzymatic inhibition by the in vitro method, and the use of mice and rats in the in vivo method. Azoxymethane, a metabolite of dimethyhydrazine (DMH), has been used extensively by many investigators to induce colon tumors and to study the effects of nutritional factors and chemopreventive agents in colon carcinogenesis. Azoxymethane (AOM) induces colon cancer in experimental animals; in a mechanism that is mediated by glutathione (GSH) depletion and impairing the total antioxidant capacity in colonic cells of rats [20,21]. In anticancer studies, researchers prefer the in vitro method, as can be seen from the larger research ratio than the in vivo method from results screened in the Scopus, Pubmed, and Google Scholar databases. A more complete explanation of the distribution of the types of methods, objects used, and natural products analyzed from 38 articles is summarized in Table 6.

## 6. Discussion and Future Directions

Cancer is the second leading cause of death in the world after cardiovascular disease. Meanwhile, colorectal cancer ranks in the top three in the number of causes of death and is ranked as the second most common cancer type in men and third in women [58,59]. A comprehensive therapy development to treat colorectal cancer is needed to reduce patient mortality. This therapy development is expected to be capable of overcoming the resistance of conventional chemotherapy agents that already exist today. Resistance of WiDr cells to 5-fluorouracil (5-FU)—an antimetabolite chemotherapeutic agent—is mediated by an increased expression of thymidylate synthetase enzyme which is the main inhibitory target of 5-FU [60,61]. WiDr is also one of the cells that have low sensitivity to treatment with 5-FU. WiDr cells are widely applicable in Indonesia due to proper carcinogenesis and tumorigenicity studies and anti-tumor testing on potency disclosure of bioactive compounds from natural products for further development. Besides, WiDr cells are identical toHCT-15 and HT-29 cell lines because they are derived from the same patient and most likely from the same tumor [61]. Overall, WiDr cells are suitable to be used as models in screening the novel compound as a co-chemotherapeutic agent with 5-FU. Combination therapy (co-chemotherapy) of 5-FU with a chemo-preventive agent is an alternative to overcome resistance, increase efficacy, and reduce adverse effects. These facts that lead the research and development of natural products become important for the future directions of medicinal plants in colorectal cancer therapy.

**Figure 2:**
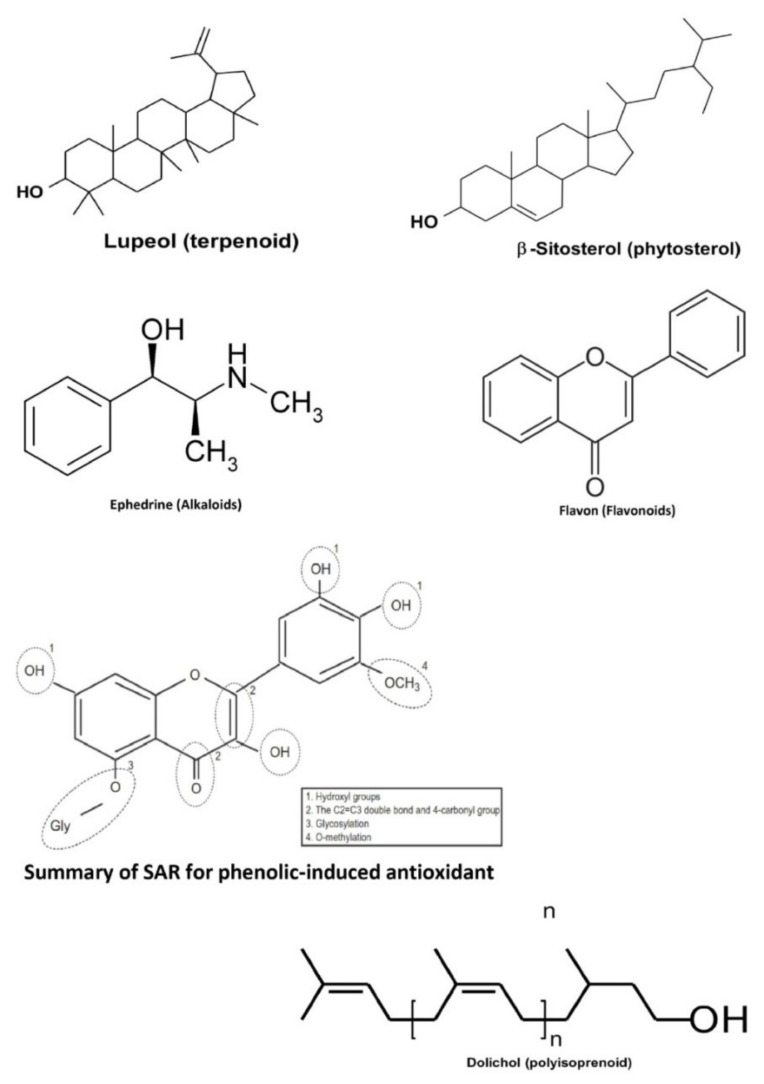
The basic structure for one of the various derivatives from bioactive compounds of terpenoids, phytosterols, alkaloids, phenolics, flavonoids, and polyisoprenoids.

Plants are one of the largest sources of natural products that are used to discover and develop novel chemotherapeutic agents. In particular, several discovered novel compounds from plants have unique mechanisms of action, greater efficacy, or lower adverse effects compared to the conventional chemotherapy drugs currently in use. Bioactive compounds from medicinal plants in Indonesia have also been studied to exhibit anticancer activities in the colorectal, namely terpenoids, phytosterols, alkaloids, phenolics, flavonoids, and polyisoprenoids (the basic structure for one of several derivatives from those bioactive compounds is presented in Figure 2).

As previously described in the research on the anti-colorectal cancer mechanism of polyisoprenoid compounds, polyisoprenoid was able to inhibit the colorectal cancer cells proliferation through up-regulation mechanisms, i.e., increased p53 gene expression (generating the cancer cell cycle blockade), induced apoptosis, and through down-regulation mechanisms, i.e., inhibition of PI3K gene expression, AKT1 gene, mTOR gene, and inhibition of EGF receptor expression [39,53]. The increase of p53 gene expression played a key role in response to cellular stress, for example, exposure to carcinogens, and also inhibited the proliferation of abnormal cells that have initiated carcinogens to prevent the development of neoplasms. The p53 gene also regulated apoptosis, inhibited angiogenesis, and regulated DNA repairment. According to the previous investigation, the increased level of the polyisoprenoid dose was able to provide a significantly increased percentage of p53 expression [48]. In addition, polyisoprenoid also has the potential to inhibit colorectal cancer cell metastasis through blockade of COX-2 expression [40,52]. It seems that colorectal cancer in Indonesia is still far beyond common understanding and more gene expression studies are needed to reveal the heterogeneous mechanisms of its process.

In the current paper, we conducted a systematic review that provided predictions for several secondary metabolites sourced from natural products to be utilized as an alternative treatment against colorectal cancer in Indonesia. They are also prospective candidates for future co-chemotherapy agents in safety, quality, standardization, and efficaciousness. This finding emphasized the potential of several natural products as anticancer agents against colorectal cancer cells.

The rich biodiversity of Indonesian plants derived from tropical rainforests provides a diversity of structural compounds that can be used as anti-colorectal cancer agents. At the same time, this review challenged Indonesian researchers of colorectal cancers to support drug-independence programs as these national products can be a part of the competitive multinational market. The utilization of Indonesia’s natural resources, which are rich in plant biodiversity, seems to be less than optimal compared to the number of traditional medicines that have been proven to have benefits and are scientifically proven. Indonesia has more than 30,000 species of plants and animals that have the potential as medicines and 9,600 species of plants and animals that are known to have medicinal properties. This potential can be developed to meet the growing demand for traditional medicines and health supplements based on natural ingredients [62,63].

Traditional medicine in Indonesia is popular with the name *Jamu*. *Jamu* is usually used as a plant that has medicinal properties and has served various Indonesian generations for centuries. The Indonesian government categorizes traditional medicinal preparations into three categories, namely *Jamu*, standardized herbal medicine, and *fitofarmaka* (phytomedicine). Herbal medicine is categorized as a traditional medicine that has been used empirically but has not yet been scientifically tested; standardized herbal medicine are those plants with medicinal properties that have been proven by preclinical testing; *fitofarmaka* is a medicinal plant that has been tested to the clinical stage. Until 2019, 23 natural products have been registered and have been proven in clinical trials. Therefore, they are categorized as fitofarmaka in Indonesia [63,64].

Herbal medicine has become a part of the culture in Indonesia. This has been done for generations. Its use in the long term has proven its safety and benefits empirically in the community. Despite having been used for centuries, herbal medicine had no place in the formal healthcare system in Indonesia before 1987. Since 1988, the Indonesian government, through the Ministry of Health, has been regulating and encouraging efforts to use scientific *Jamu* as an alternative and additional medicine. The Indonesian government has also established a body under the name Directorate of Traditional, Alternative, and Complementary of Health Care (BINA YANKES TRADKOM) [65,66,67]. However, in practice, the use of traditional medicine informal medicine is not a priority and is even avoided sometimes. This is due to the absence of guidelines, legal basis for use, and support within the Health Insurance Agency in Indonesia [68]. Another reason for this may be because the dose of the natural product to date to induce cytotoxicity is less than established chemotherapy. Therefore, there is a need to look for a highly toxic natural compound that may be conjugated to cancer antigen-specific antibodies. Designing a clinical trial to test the efficacy of adding herbal products in combination with chemotherapy is also a challenge. Many herbal compounds demonstrate antioxidant activity which contradicts the mechanism of chemotherapy, which is generally a pro-oxidant agent.

Leading the discovery is the main component of today’s early pharmaceutical research. The exploration of novel drugs for colorectal cancer therapy that has a fast progression can be a drug repurposing strategy to bypass preclinical steps that usually require laborious and resource-intensive work. In addition, it is also considered that the development of existing agents in the future can be more easily utilized by the community. For those purposes, the exploration of Indonesia’s frequently used natural resources biodiversity is the best choice. In the past, drug development is based on trial and error, so it was costly and time-consuming. Today, molecular modeling, with the aid of computer hardware and software (computational method), has reduced the risk and the process of discovery is more effective in cost and time [69].

Molecular docking is the computational simulation of a ligand binding to a receptor, which helps to predict binding molecule to the protein target to predict the affinity and activity. The aim of target discovery is the identification and validation of suitable drug targets for therapeutic intervention. Structure-based drug design (SBDD) is responsible for the toxicity and activity of a compound in the body. The SBDD utilizes information from the target protein structure to find the active site of the protein that binds to the drug compound. This is a challenge for a natural product such as *Jamu* which is generally not a pure compound. There is a need to purify and synthesize specific compounds that are originally derived from exotic plants to conserve the environment. Based on the prediction of the active site, compounds can be designed with the expectations to bind to the target protein and have biological activity. These techniques support those novel drug developments which have specific activities by the mechanism of drug-receptor interaction. Computer-aided drug design (CAAD) helps to identify small molecules by orienting and scoring them in the active binding site of a protein [70].

In silico approaches contribute significantly to early pharmaceutical research and are especially important in target discovery and its lead. The need for timely adaptation and application of in silico approaches in pharmaceutical research has been recognized to design and discover novel active substances better than previous compounds and is expected to improve further the overall efficiency of drug discovery [71].

## 7. Conclusions

In summary, this review revealed bioactive compounds from natural products of Indonesian plants that have been researched and have potential as anticancer agents that are most commonly experienced in men and women including colorectal cancer. The general method used for the analysis of the cytotoxic activity of colorectal cancer cells is the in vitro method using the MTT assay, the most widely used cell line is WiDr. The most studied bioactive compounds that have any activity against colorectal cancer are terpenoids, phytosterols, phenolics, alkaloids, and polyisoprenoids, but other natural products may have the potential to be developed from this study.

## Figures and Tables

**Table 1:** Search string composition adopted from defined PICO with desired focus on studies from Indonesia in both English and Bahasa Indonesia.

Language	Geographical Location	Population Search Terms	Intervention Search Terms
English	Indonesia	Colorectal cancer* OR “colorectal cancer*”	Secondary metabolite* OR natural product*
Bahasa Indonesia	Indonesia	“Kanker kolon” atau kanker usus besar*	Produk bahan alam* atau metabolit sekunder*

**Table 2:** Literature search record.

No.	Database	Search String	Date of Literature Search	Search Results
1	Scopus	((Indonesia) AND (natural product OR secondary metabolite) AND (colorectal cancer OR rectum OR colon OR bowel cancer OR adenocarsinoma))	13/02/2021	291
2	PubMed	((Indonesia) AND (natural product OR secondary metabolite) AND (colorectal cancer OR rectum OR colon OR bowel cancer OR adenocarsinoma))	13/02/2021	13
3	Google Scholar English	((Indonesia) AND (natural product OR secondary metabolite) AND (colorectal cancer OR rectum OR colon OR bowel cancer OR adenocarsinoma))	13/02/2021	50
4	Google Scholar Bahasa Indonesia	((Indonesia)AND (Produk alami OR matabolit sekunder) AND (Kanker kolorektal OR kanker usus besar OR usus besar OR kolorektal OR kanker rektum))	13/02/2021	50

**Table 3:** List of questions used for studies inclusion and exclusion during combined title and abstract, and full-text screenings.

Screening Stages	Questions	Screening Outcome
Title and abstract screening	Is the study located in Indonesia and is the natural resource from Indonesia? Does the study focus on colorectal cancer? Does the study present an assessment of natural products?	Studies are included if satisfy all questions
Full-text screening	Does the study present an anticancer agent? Does the study present any dataset related to colorectal cancer? Does the study consider cytotoxic activity? Does the study consider a compound target of natural products? Does the study contain positive/negative control? For included studies, additional following open questions are given to identify general information of the studies: Publication type (e.g., J: journal article, P: proceeding conference, T: thesis, B: book chapter, R: report) What is the type of study? (L: Laboratory, R: review, RS: remote sensing, O: Opinion) What datasets presented in the study are relevant to review?	Studies are included if satisfy at least two screening questions

**Table 4:** The trend of publication on natural products in colorectal cancer in Indonesia: (**a**) year vs the number of publications; (**b**) types of publications reported.

Year	Number of Publication ^(a)^
1990	1
2007	1
2008	2
2012	2
2013	1
2015	2
2016	4
2017	5
2018	8
2019	7
2020	5
**Types of Publication**	**Publication Reported ^(b)^**
Book/Book Chapter	1
Journal Article	35
Thesis	2

**Table 5:** Systematic review results summarized from 38 publications: (**a**) types of natural product; (**b**) the number of used cell lines; (**c**) colorectal cancer cytotoxicity analysis method.

Types of Natural Product	Number of Natural Product Reported ^(a)^
Phenolic	8
Phytosterol	9
Carotenoid	1
Terpenoid	17
Alkaloid	8
Flavonoid	5
Peptide	3
Polyketide	2
Polyisoprenoid	5
Carbolyc acid	1
Fatty acid	5
Glycoside	2
Aromatic compound	1
**Types of Cell Lines**	**Number of Used Cell Lines ^(b)^**
HCT-15	3
Colo205	1
HT-29	5
CaCo-2	2
HCT-116	6
SW-480	1
CRC	2
Colo320DM	1
WiDr	16
ADC	1
AOM CRC Rat Model	2
**Colorectal Cancer Cytotoxicity Analysis Method**	**Number of Used Method ^(c)^**
MTT in vitro assay	22
In vivo	3
Others in vitro	8

**Table 6:** Systematic review results summarized from 38 publications.

Types Method	Colorectal Cancer Cytotoxic Analysis Method	Types of Object/CRC Cell Lines	Types of Natural Products	The Concentration of the Tested Samples	IC50 Value / % Cell Viability / % Inhibition	Cytotoxicity Categorize [22]	Mechanism of Actions
In vivo	Colonic lesions induced by azoxymethane (AOM)	Rats	Non-nutritive compounds in fruits, vegetables, and fruits [23]	―	―	―	Control of cell proliferation in ACFs and/or normal-appearing crypts of rats exposed to AOM [23].
		Rats	Ethanol extract of *Phaleria macrocarpa* fruits (mostly flavonoids contains) [24]	―	―	―	The crude ethanolic extract of *P. macrocarpa* had high antioxidant activity and it modulated the oxidative stress as proved by the up-regulation of glutathione-*s*-transferase and superoxide dismutase [24].
		Rats	Water extract of *Premna oblongifolia* Merr. Leaves (polyphenolic compound) [25]	―	―	―	Natural dietary fiber and antioxidant sources (as found in fruits, vegetables, and plant extracts) may exhibit a protective effect against CRC [25].
	Xenograft model nude mice (carrying HCT-15 cells) [26]	Mice	Lissoclibadins (polysulfur aromatic alkaloids) from *Ascidian lissoclinum* [26]	―	―	―	Lissoclibadin 1 suppressed tumor growth in nude mice. Lissoclibadin 1 induced cell death via apoptosis due to the mitochondrial cytochrome dependent activation (intrinsic pathway) of the caspase-9 and caspase-3 cascade pathway [26].
In vitro	MTT assay [27,28,29,30,31,32,33,34,35,36,37,38,39,40,41,42,43,44,45,46,47]; MTS assay [48,49,50]; WST assay [26]; SRB assay [51]; apoptosis with double staining method [34,45,47,52]; cell cycle analysis [53] gene expression analysis [36,37]; mitochondrial membrane potential ѱm (MMP), cytochrome c release analysis and NFkB translocation [49]; caspase activity and inhibitor assays [49]; protein extraction, protein array and western blotting analyses [49]; computational molecular docking and statistical analyses [45,50]	HT-29 [24,49,50,54]; HCT-15 [26,27,43]; Colo205 [48]; WiDr [28,30,31,32,34,36,37,38,39,40,41,42,44,45,53]; HCT-116 [29,33,35,46,47,51]; CaCo-2 [49]	Catechin 7-*O*-apiofuranoside and didesmethyl tocotrienol [24]	12.5–200 µg/mL	68% (inhibition)	Not classified	The fraction of *P. macrocarpa* exhibited the highest activity as anti-proliferative against HT-29 cells. The compounds had antioxidant activity leading to a cytoprotective effect. The mechanisms of this chemoprevention included up-regulation of Bax and proliferation-promoting proteins (PCNA) [24].
			Lissoclibadins (polysulfur aromatic alkaloids) from *Ascidian lissoclinum* [26]	5 μM	4.0 μM	Significant / strong	Lissoclibadin 1 exerted the most potent cytotoxic effects and mainly promoted apoptosis through an intrinsic pathway with the activation of a caspase-dependent pathway in HCT-15 cells [26].
			Leptoclinidamide and (−)-leptoclinidamine from *Leptoclinides dubuis* [27]	0 to 27 μM	The compounds not displayed activity against cell line	Not classified	Not active against cancer cell line [24,26].
			Curacyclin A and B from the latex of *Jatropha curcas* L. [48]	1–1000 µg/mL	The compounds did not have any effect on cell line	No cytotoxicity	
			The ethanol extracts from 95 ascidians collected at North Sulawesi, Indonesia; shermilamine B and kuanoniamine D [43]	0 to 27 μM	6.7 µM (shermilamine B) and 4.1 μM (kuanoniamine D)	Significant / strong	Shermilamine B and kuanoniamine D was classified in the pyridoacridine alkaloids, which have been known to exhibit various bioactivities such as cytotoxicity, inhibition of topoisomerase II, anti-HIV activity, Ca^2+^ releasing activity, and intercalation with DNA [43].
			Crude ethyl acetate extract of endophytic fungi isolated from *Annona muricata* leaves; alkaloid compounds [28]	25; 50; 100; 200; 400 µg/mL	20.80 µg/mL	Moderate	Alkaloid compounds in endophytic fungal extract of isolate Sir-SM2 had a high cytotoxic effect on the colon cancer cell and the lowest toxicity to normal cells compared with other fungal extracts. The compounds have an alkylating activity that can cause breakage and damage of DNA strands, leading to the cancer cells death [28].
			Fungi derived from the marine sponge *Neopetrosia chaliniformis* [38], *Acanthostrongylophora ingens* [44], *Aspergillus nomius* NC06 [47]	100 ppm [38]	70.31% (cell viability) [38]	Not classified	Marine-derived fungus NC06 from sponge *N. chaliniformis* AR-01 showed the most selective cytotoxicity against the WiDr cell line compared to the Vero cell line [38].
				100 µg/mL [44]	12.88% (cell viability) [44]	Strong cytotoxicity (≤ 50%)	Not presented
				100; 10; 1; 0.1 µg/mL [47]	5.28 µg/mL [48]	Significant / strong	Not presented
			Polyisoprenoids (polyprenol and dolichol) from *Nypa fruticans*, *Rhizophora mucronata*, *Ceriops tagal*, *Avicennia alba, Avicennia marina* and *Avicennia lanata* leaves [31,32,39,40,41,53]	15.625; 31.25; 62.50; 125; 250; 500 μg/mL [41]	276 µg/mL (*C. tagal*) and 278 µg/mL (*R. mucronata*) [41]	No cytotoxicity	Polyisoprenoids induced apoptosis in the early-apoptosis phase and caused cell cycle arrest in the G0-G1 stage while decreasing the expression of Bcl-2 and cyclin-D1. In addition, the polyisoprenoid had a SI value for classification as highly selective and enables the suppression of COX-2 expression in WiDr cells [39,40,41].
				15.625; 31.25; 62.525; 125; 250; 500 µg/mL [31]	180.2 µg/mL (*N. fruticans*) [31]	Low	
				15.625; 31.25; 62.525; 125; 250; 500 μg/mL [32]	180.186 μg/mL (*N. fruticans*) [32]	Low	
				500; 250; 125; 62.5; 31.25 µg/mL [39]	154.987 µg/mL (*A. marina*) and 305.928 µg/mL (*A. lanata*) [39]	Low and no cytotoxicity	
				500; 250; 125; 62.5; 31.25; 15.625 µg/mL [40]	173.775 μg/mL (*A. alba*) [40]	Low	
			Ethyl acetate extract from *Trichoderma reesei* strain TV221 (EAFTrR) associated with marine sponge: *Stylissa flabelliformis* [41]	2000, 1000, 500; 400; 300; 250; 200; 150; 125; 100; 75; 62,5; 50; 25 µg/mL	88.88 µg/mL	Low	The extract has the potential of having anti-cancer genes through the capability to spur apoptosis. The mechanism of inhibition of cancer cell growth may go by cell cycle arrest, cell cycle delay, or apoptotic mechanism [42].
			Alpinumisoflavone from *Erythrina poeppigiana* [45]	100.0; 50.0; 25.0; 12.5; 6.25; 3.25 μg/mL	5.63 µg/mL	Significant / strong	Alpinumisoflavone is a flavonoid that has a pyran ring as pyranisoflavonoid. The presence of hydroxyl group in A-ring in position 5 increase the cytotoxic activity of flavonoids. The presence of hydroxyl group in B-ring in positions 4’ is shown to increase the cytotoxicity of flavonoids [45].
			Dichloromethane extract of *Canna indica* rhizomes [30]	2000, 1500, 1000, 750; 500; 250; 125 ppm	361.83 ppm	No cytotoxicity	The extract contained a compound that could induce apoptotic activity and cell cycle in the WiDr cells [30].
			Nine lichen species from six different locations in East Java, Indonesia [34]	1024, 512; 256; 128; 64; 32 μg/mL	324 μg/mL	No cytotoxicity	Not presented
			*Arcangelisia flava* L. Merr chloroform extract [35]	50; 100; 200; 300; 400 µg/mL	121.637 µg/mL	Low	The chloroform extract of *A. Flava* was capable to trigger apoptosis in the WiDr cells [36].
			*Piper crocatum* Ruiz & Pav ethanol extract [37]	1; 10; 100; 500; 1000, 2000, 4000 µg/mL	727 μg/mL	No cytotoxicity	The ethanol extract of *P. crocatum* had an activity to induce apoptosis and suppress COX-2 protein expression in WiDr cells [37].
			Peptides from *Platycephalus fuscus* [50]	0.005 mg protein/mL	91.04% (inhibition)	Not classified	The further cell-based study is essential to observe the mechanistic pathways and structure or function relationship of peptides in stimulating apoptosis [50].
			Cycloart-24-ene-26-ol-3-one from *Aglaia exima* leaves [49]	0.39–200 μM	2.4 µM (HT-29); 5.6 µM (CaCo-2)	Significant / strong	It is bound to tumor necrosis factor-receptor 1 (TNF-R1) leading to the initiation of caspase-8 and, through the activation of Bid, in the activation of caspase-9. This activity causes a reduction in mitochondrial membrane potential (MMP) and the release of cytochrome-C. The activation of caspase-8 and -9 both acts to commit the cancer cells to apoptosis through downstream caspase-3/7 activation, PARP cleavage and the lack of NFkB translocation into the nucleus [49].
			Seaweeds (extracted in four kind of organic solvents): *Gracilaria verrucose* [55]; *Ulva luctuca* and *Eucheuma cottonii* [33]; *Eucheuma Sp*. [46]	200; 100; 50; 25; 12.5; 6.25; 3.125; 1.5625 μg/mL [55]	43.9 μg/mL (*G. verrucose*) [55]	Moderate	Not presented
				51.2; 25.6; 12.8; 6.4; 3.2; 1.6; 0.8; 0.4 µg/mL [33]	69.3 μg/mL (*U. luctuca*) and 21.4 μg/mL (*E. cottonii*) [33]	Low and moderate	
				51.2; 25.6; 12.8; 6.4; 3.2; 1.6; 0.8; 0.4 μg/mL [46]	16.82 μg/mL (*Eucheuma Sp*.) [46]	Significant / strong	
			2-*O*-β-glucopyranosil cucurbitacin D, isolated from the ethyl acetate soluble fraction of Benalu batu (*Begonia sp.*) [29]	6.25; 12.5; 25; 50 μg/mL	0.002 μg/mL and 6.88% (cell viability)	Significant / strong	The presence of cucurbitacin type triterpenoid could be a marker compound for Begonia plant species. It exhibited potent cytotoxic activity against HCT-116 via apoptosis induction with a significant percentage of early and late apoptosis [29].
			Chloroform fraction of *Garcinia mangostana* fruits hulls [51]	0.01–100 μM	15.8 µM	Moderate	The chloroform fraction contained bioactive compounds that induced significant antiproliferative and cytotoxic potentials via induction of apoptosis and cell cycle arrest at G0/G1-phase, necrosis, and apoptosis in HCT-116 cells [51].
			Polygonumins A from *Polygonum minus* [35]	100; 50; 25; 12.5; 6.25; 3.13 µg/mL	3.24 µg/mL	Significant / strong	The sugar moiety, a sucrose unit, was recognized to be critical to the topoisomerase inhibition activity as antitumor drugs [35].
			*Gyrinops versteegii* (Gilg.) Domke leaves extract (chloroform and ethanol solvents). The most abundant compounds detected in both extracts were fatty acids, namely palmitic acid, stearic acid, and pentadecanoic acid [55]	―	Not determined	―	Not presented (the first reported study on metabolite profiling of *G. versteegii* leaves extract, the result supported further study on *G. versteegii* as the anticancer-resource plant)
			(*S*)-2-hydroxy-3-(octanoyloxy)propyl tetracosanoate, (*S*)-3-(((*S*)-11-acetoxy octadecanoyl)oxy)propane-1,2-diyl diacetate, docosanedioic acid, 2,5-dimethylnonadecane, lupeol, stigmasterol, b-sitosterol, heptadecanoic acid, hexanedioic acid, 1,6-bis[(2*R*)-ethylhexyl] ester, and 1,3-di-*O*-[2′,2′-di- (*p*-phenylene)] were isolated from the leaves of *Garcinia daedalanthera* Pierre, collected from Indonesia [56]	Not displayed	19.2 μM (lupeol)	Moderate	Not presented
			Fulvoplumierin; allamcin; allamandin; 2,5-dimethoxy-*p*-benzoquinone; plumericine; and lignan liriodendrin (from bark or *Plumeria rubra*) [57]	Not displayed	0.1 µg/mL (plumericine); 0.3 µg/mL (allamcin and allamandin); 1.3 µg/mL (fulvoplumierin); 1.4 µg/mL (2,5-dimethoxy-*p*-benzoquinone); 16 µg/mL (liriodendrin)	Significant / strong	Not presented

## Data Availability

No additional data is available for this paper.
